# Complete chloroplast genome sequence of *Schima superba* (Teaceae)

**DOI:** 10.1080/23802359.2021.1985405

**Published:** 2021-12-22

**Authors:** Mingzhu Zhang, Xingzhuang Ye, Sai Zhang, Yipeng Liu, Shipin Chen, Huihua Fan, Bao Liu, Guo-fang Zhang

**Affiliations:** a College of Forestry, Fujian Agriculture and Forestry University, Fuzhou, China; b Fujian Research Institute of Forestry, Fuzhou, China

**Keywords:** *Schima superba*, chloroplast genome, phylogeny, Teaceae

## Abstract

*Schima superba* is the dominant species of subtropical evergreen broad-leaved forest which has the characteristics of ecological fire prevention function. In this study, we report the complete chloroplast genome sequence of *S. superba*. The cp genome was 157,205 bp in length with a GC content of 37.40%, including a large single-copy (LSC 87,161 bp), a small single-copy (SSC 18,092 bp), and a pair of inverted repeats (IR 25,976 bp). The genome encoded 133 functional genes, including 88 protein-coding genes, 37 tRNA genes, and 8 rRNA genes. The phylogenetic analysis showed that *S. superba* was closely related to *Schima sinensis*, *Schima multibracteata*, *Schima crenata*, and *Schima remotisertata*.

*Schima superba* Gardn. et Champ. (Teaceae), an evergreen broad-leaved tree species, which has the characteristics of rapid growth, high yield, excellent timber and strong adaptability. It is the dominant species of subtropical evergreen broad-leaved forest in China and widely distributed in the southern of China (Yao et al. 2017). *Schima superba* can not only be used as an excellent wood, but also can be used for medical treatment, and landscaping. Compared with other tree species, *S. superba* has the advantage of ecological fire prevention function, and is listed as one of the important and high-quality tree species for afforestation in southern China (Zhang et al. 2013). In recent years, as more and more data have been uploaded to the NCBI GenBank, it has provided more information on the phylogeny of *Schima superba*. Here, we report the complete chloroplast genome sequences of *S. superba*, and reveal the phylogenetic relationships to related species in Theaceae.

The sample of *S. superba* was collected from FuJian Province, China (Fujian Agriculture and Forestry University, Fuzhou: 26°04′49.51″N, 119°14′23.33″E). A specimen was deposited in the Herbarium of College of Forestry, Fujian Agriculture and Forestry University (Bao Liu, liubao@m.fafu.edu.cn) under the specimen number: FAFU0723. The improved CTAB method (Doyle 1987) was used to extract DNA of the sample. DNA was sent to BGI (The Beijing Genomics Institute) to construct DNA library and sequenced by MGISEQ-2000RS platform, with approximately 7 GB of data generated. The chloroplast genome of *S. superba* was then assembled using the GetOrganelle pipe-line (https://github.com/Kinggerm/GetOrganelle), by recruiting plastid-like reads. Final reads were viewed and edited by Bandage (Wick et al. 2015). The assembled chloroplast genome annotation was based on the comparison with *Schima crenata* by Geneious v.11.1.5 (Kearse et al. 2012). The annotation results were drawn with the online tool OGDRAW (http://ogdraw.mpimp-golm.mpg.de/) (Marc et al. 2013). After the accomplishment by annotating, the Sequin file was output and submitted to the NCBI database to obtain the GenBank accession.

The complete chloroplast genome sequence of *S. superba* (GeneBank accession: MZ475301) was 157,205 bp in length with a GC content of 37.40%, including a large single-copy (LSC) region of 87,161 bp, a small single-copy (SSC) region of 18,092 bp, and a pair of inverted repeats (IR) regions of 25,976 bp. The complete chloroplast genome contains 133 genes, with 88 protein-coding genes, 37 tRNA genes, and 8 rRNA genes.

In order to reveal the phylogenetic position of *S. superba*, a phylogenetic analysis was performed based on 29 species of Theaceae and 2 species from Symplocaceae as outgroup, the date was downloaded from NCBI GenBank. The sequences were aligned using PhyloSuite (Zhang et al. 2020). A maximum-likelihood (ML) tree was constructed based on the 86 complete chloroplast genome sequences using the CIPRES Science Gateway web server (RAxML-HPC2 on XSEDE 8.2.12) (Miller et al. 2010) with 1000 bootstrap replicates. The phylogenetic tree was visualized using FigTree v1.4.3 (Rambaut 2016). The phylogenetic tree showed that *S. superba* was most closely related to *Schima sinensis*, *Schima multibracteata*, *Schima crenata*, and *Schima remotisertata* (Figure 1).

**Figure 1.**
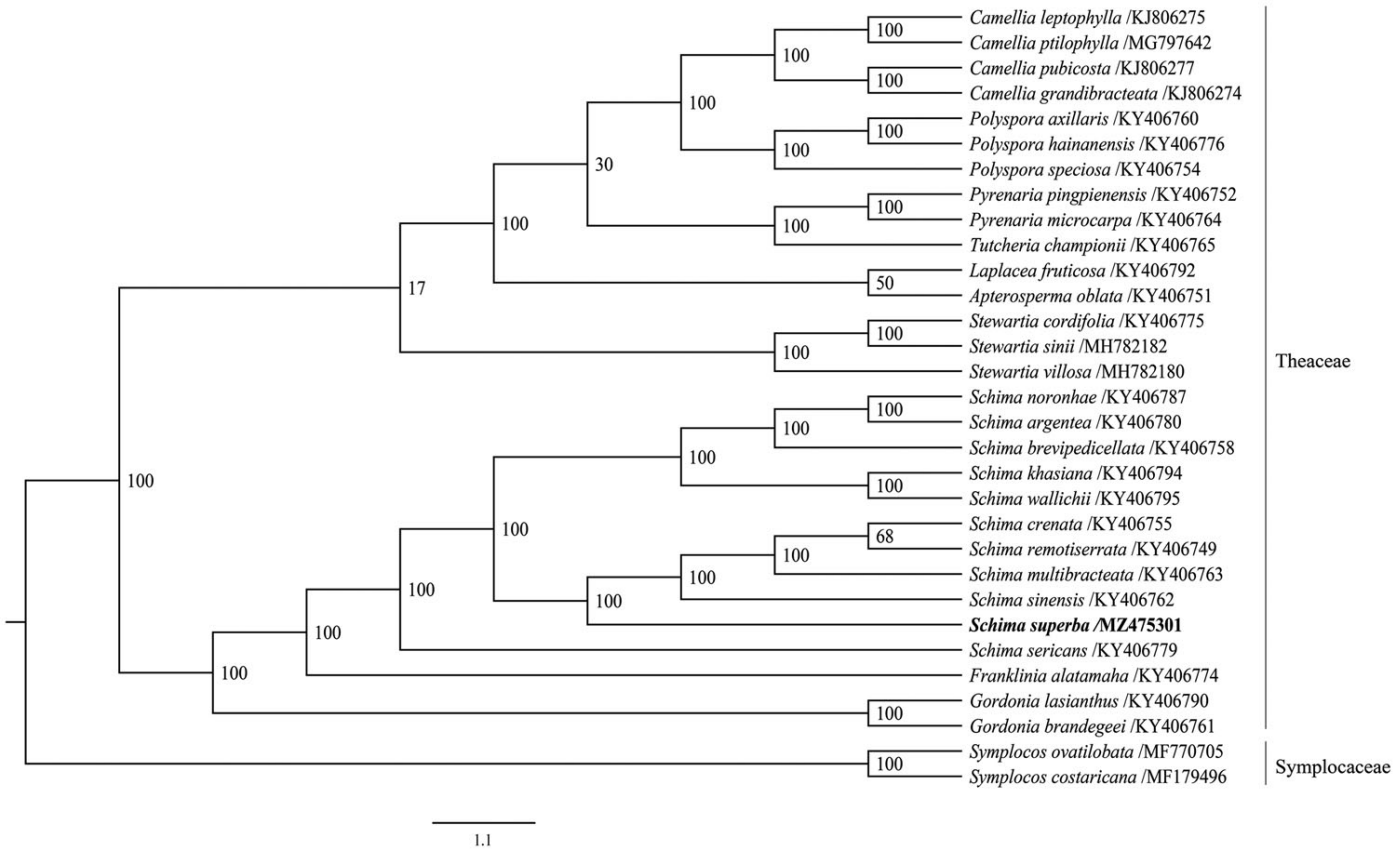
Maximum likelihood phylogenetic tree of 29 species of Theaceae and 2 species from Symplocaceae as outgroup based on complete plastome sequences. Number on the right of nodes showed the bootstrap value.

## Data Availability

The genome sequence data that support the findings of this study are openly available in GenBank of NCBI at [https://www.ncbi.nlm.nih.gov] under the accession no.MZ475301. The associated BioProject, SRA, and Bio-Sample numbers are PRJNA743231, SRX11369043, and SAMN20003101 respectively.
